# Current infection control practices for multidrug-resistant organisms (MDRO): a survey of the Society for Healthcare Epidemiology of America (SHEA) research network and affiliated US-based hospitals

**DOI:** 10.1017/ice.2025.10394

**Published:** 2026-04

**Authors:** K.C. Coffey, Trudy Grossman, David B. Banach, Anthony D. Harris, David C. Hooper, Susan S. Huang, Erica S. Shenoy

**Affiliations:** 1 Massachusetts General Hospital and Harvard Medical School, Boston, MA, USA; 2 Combating Antibiotic-Resistant Bacteria Biopharmaceutical Accelerator (CARB-X), USA; 3 University of Connecticut School of Medicine, USA; 4 University of Maryland School of Medicine, USA; 5 University of California Irvine School of Medicine, USA; 6 Mass General Brigham Inc., USA

## Abstract

**Objective:**

To understand current practices for screening, implementing and clearing contact precautions (CP) for multidrug-resistant organisms (MDRO).

**Design:**

A survey of US-based SHEA Research Network (SRN) members and Community Healthcare Epidemiologists and Stewards (CHES).

**Participants:**

The SRN is a consortium of healthcare facilities collaborating on multicenter research projects. CHES is an SRN-affiliated group of community hospital epidemiologists.

**Methods:**

A Research Electronic Data Capture (REDCap) survey was administered between January 7, 2025 and February 25, 2025. Frequencies and proportions were calculated in REDCap and Microsoft Excel. Fisher’s exact and *χ* ^2^ tests were calculated in *R*.

**Results:**

Of 134 facilities, 60 (45%) responded; 4 answered only demographic questions and were excluded from analysis. Most facilities (70%) performed active surveillance for ≥1 MDRO, but only 16% employed preemptive CP. All respondents reported using CP for ≥1 MDRO. CP were employed more often for infection than colonization. Clearance protocols to discontinue CP were common for MRSA (97%), *C. difficile* (95%), and vancomycin-resistant enterococci (82%), but uncommon for Gram-negatives. Training and adherence of frontline staff (70%), unavailability of private rooms (41%), and lack of evidence-based strategies to eradicate reservoirs (34%) were the top 3 identified barriers to MDRO infection control and prevention.

**Conclusions:**

Infection control practices varied by infection, colonization, specific MDRO, and time to clearance. Variation may indicate need for more data on transmission risk by disease state, organism, and time. Evidence-based strategies are needed to guide MDRO prioritization and CP duration and clearance policies. Guidance should acknowledge limitations in adherence, availability of private rooms, and persistent environmental reservoirs as major barriers to MDRO containment identified in this survey.

## Introduction

Colonization with multidrug-resistant organisms (MDRO) is associated with increased risk of infection and transmission in healthcare facilities.^1–3^ Screening, implementation, and clearing of contact precautions (CP) for MDRO in healthcare settings is heterogenous and not well-described in the United States. Practices may vary by facility type, location, size, and patient population.^4–6^

A 2012 survey of the Association for Professionals in Infection Control and Epidemiology (APIC) evaluated policies for discontinuing CP for methicillin-resistant *Staphylococcus aureus* (MRSA) and vancomycin-resistant enterococci. Of those surveyed, 72.6% and 56.5% reported institutional policies allowing for discontinuation of CP for MRSA and vancomycin-resistant enterococci, respectively.^7^ The combination of time- and culture-based criteria for discontinuation of CP yielded 64 distinct strategies for MRSA and 48 unique strategies for vancomycin-resistant enterococci highlighting the substantial variability in CP discontinuation policies. The 2014 SHEA Research Network (SRN)^8^ survey aimed to assess definitions and infection control practices for multidrug-resistant Gram-negative bacteria.^9^ That survey showed variability in the definition of “multidrug-resistant“ (MDR). Also, whereas CP were commonly used for MRSA, vancomycin-resistant enterococci and carbapenem-resistant Enterobacterales (CRE), 20% of facilities did not use CP for MDR *Pseudomonas aeruginosa* (MDR-PsA) or MDR *Acinetobacter baumannii* (MDR-AB). Most facilities that isolated for MDRO also cleared precautions according to a wide array of criteria.

In 2014, Weiner et al. investigated MDRO infection control policies among US healthcare facilities using data from the National Healthcare Safety Network Annual Facility Survey. Their survey found that 80% of acute care hospitals reported use of CP for patients infected or colonized with MRSA, CRE, or vancomycin-resistant enterococci, and 70% reported a CP policy for extended-spectrum *β*-lactamase producing Enterobacterales (ESBL). These data varied by geographic region and facility type as did reported active surveillance (AS) practices.^6^

As these surveys took place over a decade ago, this survey sought to assess the current practices of US-based facilities by soliciting input from healthcare epidemiologists and infection control programs on screening, implementation, and clearance of CP, along with perceived barriers to MDRO infection prevention.

## Methods

The survey tool used in this study (Supplement 1) was developed by KC, ESS and TG, extrapolated from a scoping review of similar nation-wide surveys of MDRO screening practices in the literature,^1,5,7,9–11^ updating prior surveys.^6,12^ Demographic data were collected in accordance with the American Hospital Association annual survey.^13^ Included MDRO were selected from the CDC’s 2019 Antibiotic Resistance Threats Report^3^ and the most recent update describing the seven most common antimicrobial-resistant pathogens found in healthcare settings.^14^ Targeted MDRO included: MRSA, vancomycin-resistant enterococci, ESBL, CRE, MDR-PsA, carbapenem-resistant *Pseudomonas aeruginosa* (CR-PsA), MDR-AB, carbapenem-resistant *Acinetobacter baumannii* (CRAB), and *Clostridioides difficile*. *Candidozyma auris* was not explicitly queried owing to a concomitant *C. auris*-specific SRN survey.^15^ The draft survey was reviewed and revised by subject matter experts (DB, AH, DH, SH) through an iterative process. The 54-item questionnaire was primarily categorical, including ranked responses and Likert-scale responses. The survey also applied branching logic to ask more detailed questions on endorsed practices.

We surveyed 134 US-based healthcare facilities participating in the SRN and SHEA Community Healthcare Epidemiologists and Stewards (CHES) on current practices for surveillance, implementation, and removal of CP. CHES is an SRN-affiliated group of community hospital epidemiologists whose inclusion provided greater representation of smaller, community hospitals compared to the predominantly large academic facilities represented in the broader SRN. The survey was administered online January 7, 2025-February 25, 2025. Reminders were auto generated and sent to nonresponding sites weekly until survey close. Although no financial compensation was provided to participants, respondents were incentivized by a raffle to receive one of five $100 gift cards upon survey completion.

Study data were collected and managed using Research Electronic Data Capture (REDCap) tools hosted at MGB.^16,17^ Duplicate responses from a single facility were excluded. Responses were de-identified and not linked to individuals or facilities. Frequencies and proportions were calculated in REDCap and Microsoft Excel. Fisher’s exact and χ^2^ tests were performed using base *R* (version 4.5.1). This survey was considered nonhuman subjects research by the Mass General Brigham Institutional Review Board.

## Results

Of 134 facilities, 60 (45%) submitted the survey; 4 answered only demographic questions and were excluded from analysis. Participating facilities were from 25 US states, and 91% were in cities, 9% were in towns; there were no facilities that described themselves as rural (Table 1).

**Table 1.** Demographics of 56 respondent facilities

Variable	No. (%)^*^
**Census division**	
New England	11 (20%)
Middle Atlantic	9 (16%)
East North Central	7 (13%)
Pacific	7 (13%)
West North Central	4 (7%)
East South Central	3 (5%)
West South Central	2 (4%)
Mountain	2 (4%)
No response	11 (20%)
**Affiliation**	
SHEA Research Network	41 (73%)
SHEA Community Healthcare Epidemiologists and Stewards	5 (9%)
No response	10 (18%)
**Location**	
City	51 (91%)
Town	5 (9%)
Rural	0 (0%)
**Entity**	
Non-government, not-for-profit	43 (77%)
Government, non-federal	9 (16%)
Investor-owned, for-profit	2 (4%)
Government, federal	2 (4%)
**Teaching Institution**	
Yes	49 (88%)
No	7 (13%)
**Size**	
Large (500+ Beds)	30 (54%)
Medium (100–499 Beds)	24 (43%)
Small (1–99 Beds)	2 (4%)
**Critical access**	
Yes	9 (16%)
No	47 (84%)
**Facility has an ICU**	
Yes	56 (100%)
No	0 (0%)
**Pediatric only or pediatric predominant**	
Yes	7 (13%)
No	49 (88%)
**No. annual admissions**	
50,000 or more	8 (14%)
10,000–49,999	21 (38%)
5,000–9,999	4 (7%)
1,000–4,999	5 (9%)
Less than 1,000	0 (0%)
No response	18 (32%)
**Room types**	
Predominantly single occupancy	33 (59%)
Predominantly double occupancy	7 (13%)
Mix of multiple shared room types	16 (29%)

* Percents are rounded and may not add to 100%

### MDRO practices

**Figure 1.**
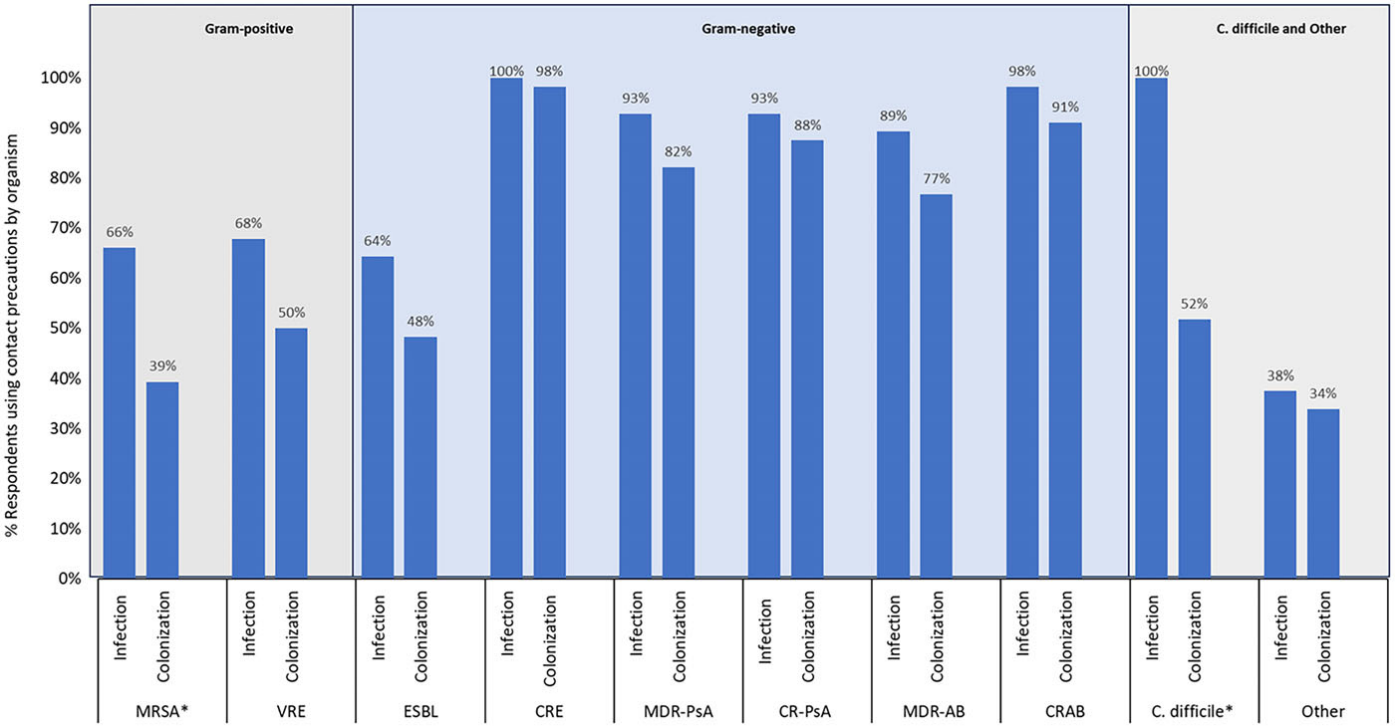
Use of contact precautions by organism and infection versus colonization status, *n* = 56. *Statistically more likely to use CP for infection versus colonization (*P* < .05). MRSA, methicillin-resistant *Staphylococcus aureus;* VRE, vancomycin-resistant Enterococcus; ESBL, extended-spectrum *β*-lactamase producing Enterobacterales; CRE, carbapenem-resistant Enterobacterales; MDR-PsA, multidrug-resistant *Pseudomonas aeruginosa;* CR-PSA, arbapenem-resistant *Pseudomonas aeruginosa;* MDR-AB, multidrug-resistant *Acinetobacter baumannii;* CRAB, carbapenem-resistant *Acinetobacter baumannii; C. difficile, Clostridioides difficile*.

**Figure 2.**
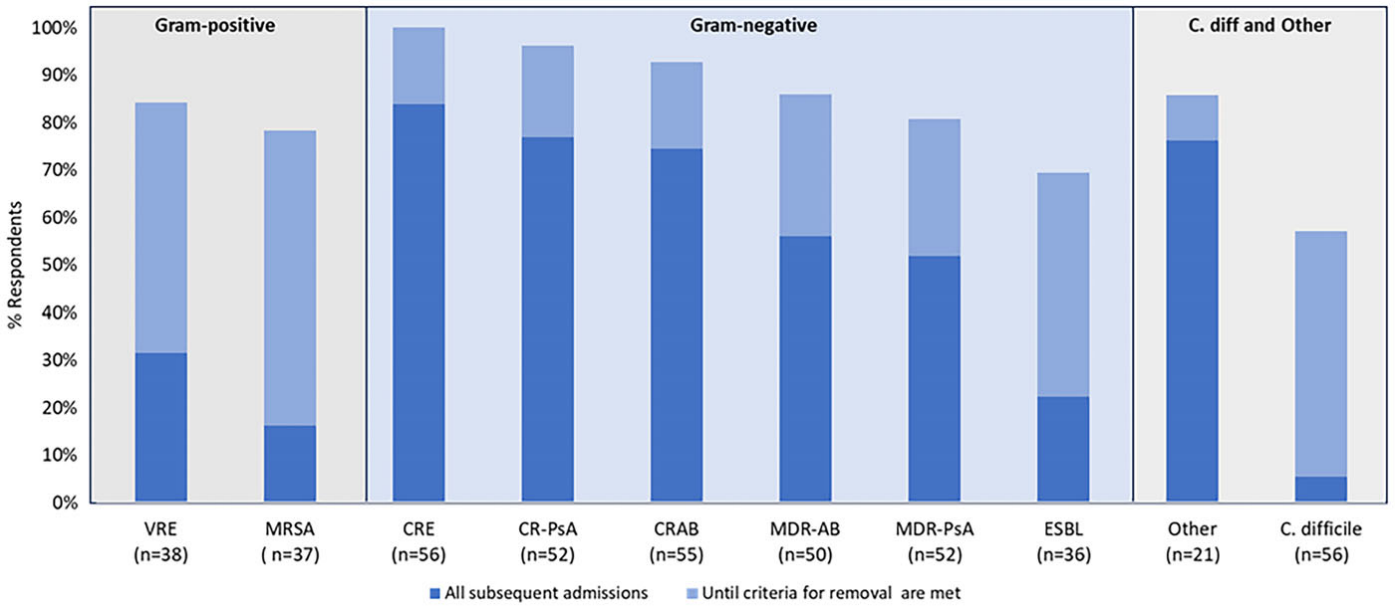
If contact precautions implemented initially, % reinstituted on readmission by MDRO. MRSA, methicillin-resistant *Staphylococcus aureus;* VRE, vancomycin-resistant Enterococcus; ESBL, extended-spectrum *β*-lactamase producing Enterobacterales; CRE, carbapenem-resistant Enterobacterales; MDR-PsA, multidrug-resistant *Pseudomonas aeruginosa;* CR-PSA, carbapenem-resistant *Pseudomonas aeruginosa;* MDR-AB, multidrug-resistant *Acinetobacter baumannii;* CRAB, carbapenem-resistant *Acinetobacter baumannii; C. difficile, =Clostridioides difficile*.

All facilities reported using CP for at least 1 MDRO, calculated independently for infection and colonization. In general, facilities employed CP more often for infection than colonization when comparing the same organism, and more often for Gram-negative compared to Gram-positive organisms (Figure 1). Respondents were statistically more likely to use CP for infection versus colonization for MRSA (*P* = .005) and *C. difficile* (*P* < .001). Vancomycin-resistant enterococci trended toward statistical significance (*P* = .05). For all Gram-negative organisms, the difference between reported use of CP for infection versus colonization was decidedly smaller and not statistically significant (*P* > .05). When CP were instituted, they were more often applied to all patient populations rather than by specific patient population. Of facilities who employ CP for a given MDRO, >85% do so for all patients rather than by patient population. Specifically, CP are used for all patients in 86% of the 37 sites who use CP for MRSA, 94% of the 36 sites who use CP for vancomycin-resistant enterococci, and 100% of the 56 sites for CRE. On average across all Gram-negative organisms, among facilities that applied CP, 63% reported continuing precautions on all subsequent admissions, although this practice varied by organism (Figure 2). When asked about any additional measures utilized to prevent MDRO transmission, signage was the most common across all organisms (41%) followed by enhanced room cleaning after discharge (28%). Cohort healthcare staff was rare (1%) across all MDRO.

MRSA, *C. difficile,* and ESBL were the top three MDRO reported as common or very common; CRAB was reported as rare by 52/56 (93%) of respondents. Facilities identified *C. difficile* (37/56, 66%), CRE (34/56, 61%), and ESBL (23/56, 41%) as the most concerning. Respondents described lack of training/adherence to CP of frontline staff, unavailability of private rooms, and lack of evidence-based strategies to eradicate environmental reservoirs as the top three barriers to effective MDRO infection prevention. Insufficient staffing was ranked in the top 3 barriers by about 20% of respondents (10/56). When insufficient staffing was a barrier, respondents indicated that nurses and cleaning staff were the roles most needed.

### Surveillance practices

Facilities were queried about screening practices in the context of routine surveillance rather than outbreak response. Active surveillance (AS) was defined as screening tests used to identify asymptomatic colonization for the purpose of identifying carriers. Most facilities, 39/56 (70%), performed AS for at least one MDRO. Active surveillance for MRSA was performed by 33/56 (59%) facilities versus 12/56 (21%) for CRE; some MRSA surveillance may be driven by state legislative mandate as indicated by 13 respondents. Facilities that performed AS were more likely to do so on admission to specific units (30/56, 54%) versus all admissions (13/56, 13%), at intervals throughout admission (13/56, 13%), or on discharge from a specific unit (5/56, 9%). Only 2 facilities (4%) reported performing AS for any MDRO on facility discharge. MRSA surveillance predominated in the intensive care unit (19/56, 34%). Several respondents reported performing AS for MDRO in immunocompromised patients, CRE (5), vancomycin-resistant enterococci (4), MRSA (3), ESBL (2), and *C. difficile* (2).

Most surveillance testing was reported as being processed on-site, and a majority reported turnaround times of <24 hours; the rest reported less than one week to result. Polymerase chain reaction (PCR) was the most common mode of surveillance testing, compared to culture, but this varied by MDRO. Of the sites performing AS for MRSA, 22/33 (67%) reported using PCR as opposed to culture (13/33, 39%). Of the sites performing AS for vancomycin-resistant enterococci, 7/8 (88%) reported using culture compared to 2/8 (25%) PCR. AS for Gram-negative MDRO was less common. Of the 12 sites performing AS for CRE, 10/12 (83%) reported using PCR versus 2/12 (17%) culture. Only 3 sites reported performing AS for ESBL, 2 of which reported using culture. Two sites reported performing AS for CR-PsA with 1 site using culture, the other using PCR. Two sites reported performing AS for CRAB, both via PCR. No sites reported performing AS for MDR-PsA or MDR-AB. Four sites reported performing AS for *C. difficile*, all via PCR.

### Preemptive CP implementation practices

Respondents were asked whether they utilize preemptive CP for patients without known MDRO who are admitted or transferred to their facility. Only 9/56 (16%) reported employing preemptive CP for any MDRO, predominantly for direct transfers from a facility with a known outbreak of a given MDRO. Other reported indications for preemptive CP included transfer from or recent hospitalization in a specified area, U.S—or non-U.S.—region.

### Clearance practices

When asked whether their facility has a protocol for discontinuing CP for patients with a history of any of the targeted MDRO, 54/56 (96%) indicated that they had a clearance protocol for at least one MDRO. MDRO for which facilities had clearance protocols were most commonly MRSA (97%), *C. difficile* (95%), and vancomycin-resistant enterococci (82%) (Table 2). Only 20% of facilities reported having a protocol to clear CP for CRE. Protocols were categorized as time-based and/or microbiology-based but were not mutually exclusive. For all MDRO, time was a more frequent component of the clearance protocol than microbiologic results. However, time to clearance varied widely from “end on discharge” to “more than a year” across all organisms (Table 2). Among facilities employing a microbiology-based clearance protocol, there was variation in the number of specimens required ranging from 1–3 for most MDRO.

**Table 2.** Clearance protocols to discontinue contact precautions by targeted MDRO

Facility employs CP for targeted MDRO	MRSA n = 37	VRE *n* = 38	ESBL *n* = 36	CRE *n* = 56	MDR-PsA *n* = 52	CR-PsA *n* = 52	MDR-AB *n* = 50	CRAB *n* = 55	*C. difficile* *n* = 56	Other *n* = 21
Facility has a protocol for clearing CP, *n* (%)	36 (97%)	31 (82%)	27 (75%)	11 (20%)	28 (54%)	16 (31%)	24 (71%)	14 (25%)	53 (95%)	5 (24%)

Types of clearance protocols employed, *n* (%)	MRSA *n* = 36	VRE *n* = 31	ESBL *n* = 27	CRE *n* = 11	MDR-PsA *n* = 28	CR-PsA *n* = 16	MDR-AB *n* = 24	CRAB *n* = 14	*C. difficile* *n* = 53	Other *n* = 5
Time only	12 (33%)	10 (32%)	16 (59%)	4 (36%)	13 (46%)	6 (38%)	11 (46%)	5 (36%)	21 (40%)	3 (60%)
Micro only	5 (14%)	8 (26%)	2 (7%)	0 (0%)	3 (11%)	2 (13%)	1 (4%)	1 (7%)	0 (0%)	0 (0%)
Other only	6 (17%)	8 (26%)	3 (11%)	2 (18%)	3 (11%)	3 (19%)	3 (13%)	3 (21%)	22 (42%)	2 (40%)
Time + Micro	11 (31%)	5 (16%)	4 (15%)	3 (27%)	4 (14%)	2 (13%)	3 (13%)	1 (7%)	1 (2%)	0 (0%)
Time + Other	2 (6%)	0 (0%)	1 (4%)	0 (0%)	3 (11%)	1 (6%)	3 (13%)	2 (14%)	7 (13%)	0 (0%)
Time + Micro + Other	0 (0%)	0 (0%)	0 (0%)	1 (9%)	0 (0%)	0 (0%)	0 (0%)	0 (0%)	0 (0%)	0 (0%)
Micro + Other	0 (0%)	0 (0%)	1 (4%)	1 (9%)	1 (4%)	1 (6%)	1 (4%)	1 (7%)	0 (0%)	0 (0%)
No response	0 (0%)	0 (0%)	0 (0%)	0 (0%)	1 (4%)	1 (6%)	2 (8%)	1 (7%)	2 (4%)	0 (0%)

Any Time-based protocol, n (%)	MRSA *n* = 25	VRE *n* = 15	ESBL *n* = 21	CRE *n* = 8	MDR-PsA *n* = 20	CR-PsA *n* = 9	MDR-AB *n* = 17	CRAB *n* = 8	*C. difficile* *n* = 29	Other *n* = 3
End on discharge	4 (16%)	2 (13%)	6 (29%)	0 (0%)	7 (35%)	0 (0%)	5 (29%)	0 (0%)	14 (48%)	1 (33%)
Less than one month	0 (0%)	0 (0%)	1 (5%)	0 (0%)	1 (5%)	0 (0%)	1 (6%)	0 (0%)	5 (17%)	0 (0%)
1–3 months	1 (4%)	2 (13%)	3 (14%)	0 (0%)	2 (10%)	0 (0%)	1 (6%)	0 (0%)	7 (24%)	1 (33%)
>3–6 months	3 (12%)	4 (27%)	3 (14%)	2 (25%)	2 (10%)	2 (22%)	2 (12%)	2 (25%)	2 (7%)	0 (0%)
>6–12 months	8 (32%)	3 (20%)	3 (14%)	2 (25%)	2 (10%)	4 (44%)	3 (18%)	3 (38%)	0 (0%)	0 (0%)
More than 1 year	8 (32%)	4 (27%)	5 (24%)	4 (50%)	6 (30%)	3 (33%)	5 (29%)	3 (38%)	1 (3%)	1 (33%)
No response	1 (4%)	0 (0%)	0 (0%)	0 (0%)	0 (0%)	0 (0%)	0 (0%)	0 (0%)	0 (0%)	0 (0%)

Any microbiology-based protocol, *n* (%)	MRSA *n* = 16	VRE *n* = 13	ESBL *n* = 7	CRE *n* = 5	MDR-PsA *n* = 8	CR-PsA *n* = 5	MDR-AB *n* = 5	CRAB *n* = 3	*C. difficile* *n* = 1	Other *n* = 0
Site^*^										
One naris	1 (6%)	0 (0%)	0 (0%)	0 (0%)	0 (0%)	0 (0%)	0 (0%)	0 (0%)	0 (0%)	0
Bilateral nares	14 (88%)	0 (0%)	0 (0%)	0 (0%)	0 (0%)	0 (0%)	0 (0%)	0 (0%)	0 (0%)	0
Oropharyngeal	1 (6%)	0 (0%)	0 (0%)	0 (0%)	0 (0%)	0 (0%)	0 (0%)	0 (0%)	0 (0%)	0
Groin	2 (13%)	0 (0%)	1 (14%)	1 (20%)	1 (13%)	1 (20%)	0 (0%)	1 (33%)	0 (0%)	0
Axilla	2 (13%)	0 (0%)	0 (0%)	0 (0%)	0 (0%)	0 (0%)	0 (0%)	0 (0%)	0 (0%)	0
Site of initial infection	6 (38%)	3 (23%)	5 (71%)	2 (40%)	7 (88%)	4 (80%)	3 (60%)	1 (33%)	1 (100%)	0
Peri-rectal/rectal/stool	0 (0%)	12 (92%)	3 (43%)	2 (40%)	1 (13%)	1 (20%)	0 (0%)	0 (0%)	0 (0%)	0
Other	3 (19%)	1 (8%)	0 (0%)	1 (20%)	1 (13%)	0 (0%)	1 (20%)	0 (0%)	0 (0%)	0
No response	1 (6%)	0 (0%)	1 (14%)	1 (20%)	0 (0%)	1 (20%)	1 (20%)	1 (33%)	0 (0%)	0
# Specimens										
1	5 (31%)	2 (15%)	2 (29%)	1 (20%)	3 (38%)	1 (20%)	2 (40%)	1 (33%)	1 (100%)	0
2	3 (19%)	1 (8%)	2 (29%)	2 (40%)	1 (13%)	1 (20%)	0 (0%)	1 (33%)	0 (0%)	0
3	6 (38%)	9 (69%)	2 (29%)	0 (0%)	3 (38%)	2 (40%)	1 (20%)	0 (0%)	0 (0%)	0
More than 3	1 (6%)	0 (0%)	0 (0%)	0 (0%)	0 (0%)	0 (0%)	0 (0%)	0 (0%)	0 (0%)	0
No response	1 (6%)	1 (8%)	1 (14%)	2 (40%)	1 (13%)	1 (20%)	2 (40%)	1 (33%)	0 (0%)	
Time between specimens										
24–48 hours	5 (31%)	3 (23%)	1 (14%)	1 (20%)	1 (13%)	1 (20%)	0 (0%)	1 (33%)	0 (0%)	0
>24 hours-1 week	3 (19%)	2 (15%)	2 (29%)	0 (0%)	2 (25%)	1 (20%)	1 (20%)	0 (0%)	0 (0%)	0
>1 week	1 (6%)	4 (31%)	1 (14%)	1 (20%)	1 (13%)	1 (20%)	0 (0%)	0 (0%)	0 (0%)	0
Other	1 (6%)	1 (8%)	0 (0%)	0 (0%)	0 (0%)	0 (0%)	0 (0%)	0 (0%)	0 (0%)	0
No response	6 (38%)	3 (23%)	3 (43%)	3 (60%)	4 (50%)	2 (40%)	4 (80%)	2 (67%)	1 (100%)	0

* Respondents could choose multiple micro-based sites, therefore % may add up to over 100%.

## Discussion

This survey updates findings from prior SRN surveys on hospital protocols for surveillance and use of CP for MDRO. It further expands on the details of target populations for AS and protocols for clearance. The data reveal a shifting trend in application of AS and CP but a persistence of heterogenous practices to discontinue CP by MDRO.

This survey showed that AS is more commonly applied to specific units rather than to all admissions. More than half of respondents reported performing AS for at least one MDRO on admission to a specific unit compared to less than a quarter on all admissions. Facilities most frequently applied AS to the ICU patient population. The practice of AS in the ICU appears to be relatively stable but preemptive CP appears to be decreasing compared to a 2008 survey. In that survey of National Healthcare Safety Network hospitals, 40% of ICU admissions were screened for at least one MDRO, and 31% employed preemptive CP pending screen results.^4^ In our survey, only 16% of facilities reported using preemptive CP. Further investigation is needed to determine the reason that preemptive CP use was low. Potential motivations include material constraints, throughput concerns, environmental impact considerations, and availability and rapid turnaround of AS tests. We surmise that a combination of these factors may contribute.

Compared to the 2012 APIC survey, clearance protocols to discontinue CP have become more commonplace, particularly for Gram-positive MDRO compared to Gram-negative MDRO. Nevertheless, wide variation persisted in the details of both time-based (amount of time) or microbiology-based (number of swabs and choice of body site) protocols to discontinue CP. In the earlier study, three-quarters of respondents indicated their facility had a policy to discontinue CP for MRSA^7^ compared to nearly all respondents in this survey; the trend was similar for vancomycin-resistant enterococci, half of respondents in 2012 compared to over 80% in this survey. In this survey, most of the reported clearance protocols included time-based criteria; fewer than half included repeated sampling for stopping CP, although some sites used a mix of both strategies. This was despite most AS being reported as performed via PCR with a turnaround time of <24 hours, making it easier and faster to screen compared to earlier surveys. Rationale for the trend toward time-based clearance may be due to increased data supporting its association with microbiologic clearance and ease of implementation, although either method of clearance can improve hospital operations and bed management.^18–20^ The trend may also indicate a pragmatic, operational approach to CP as the volume of MDRO increases.

It was notable in this survey that CP were employed more often for infection than colonization. It has been shown that both infected and colonized patients transmit MDRO.^21–25^ While patients with active infections are presumed to have a higher bacterial load and therefore shed more into the environment, colonized patients have been shown to readily and equally contribute to environmental contamination.^21^ It is possible that this shift was driven by healthcare worker fatigue of CP, as the overall number and combined prevalence of MDRO have increased. Most of the published literature, however, does not make a distinction between CP for infection versus colonization in transmission pathways.^22,23^ A growing body of evidence demonstrates an increased infection risk following colonization with an MDRO^24,25^ indicating the importance of transmission interruption.

Another interesting juxtaposition of reported practices existed between CP implementation and AS screening. When facilities reported using CP for a targeted MDRO, CP tended to be used across all patient types and locations. But AS was limited to specific patient populations, such as ICU and oncology or transplant units. In practice, this may be another example of the previously noted dichotomy of CP practices for infection versus colonization. If so, this would suggest an implicit tolerance for transmission risk in certain populations and/or a competing desire to avoid CP when able. Alternatively, this may be the result of pragmatic implementation recognizing that AS is labor- and cost-intensive and targeting AS to high-risk populations to optimize the cost-benefit ratio.

Another apparent contradiction was that MRSA was the MDRO for which the most AS was done, but fewer facilities reported implementing CP for MRSA colonization than other targeted MDRO. For example, 33 facilities reported performing AS for MRSA, but only 22 facilities reported employing CP for MRSA colonization; in contrast, 12 facilities reported AS for CRE, but 55 sites reported use of CP for known CRE-colonized patients. AS is typically associated with an intervention when screening tests are positive. The 2022 SHEA Practice Recommendation to prevent MRSA transmission proposes that hospitals without ongoing MRSA outbreaks, high or increasing rates of MRSA infection, or high or increasing rates of hospital-onset MRSA that have strong horizontal prevention measures can consider modifying the use of CP for MRSA-colonized patients and consider implementing a decolonization program while monitoring acquisition rates.^26^ This survey did not specifically inquire what other actions, if any, were taken by those facilities that perform AS for MRSA but did not report employing CP for MRSA colonization. Nor were we able to correlate higher frequency of MRSA AS with legislation mandating AS for MRSA, although 13 facilities responded that MRSA AS was mandated by legislation. Lastly, while not AS according to the survey definition, some facilities may have considered MRSA swabs used to inform antibiotic selection rather than CP which could have affected their responses.

The contradictions above may reflect facilities’ responses to perceived barriers to MDRO infection prevention. Availability of private rooms was second in the top 3 reported barriers. Where practice deviated from guidelines, eg, less frequent use of CP for colonization compared to infection, operational limitations may be the driver. This may be further shown in the survey responses about the most troubling MDRO. *C. difficile, CRE,* and ESBL were reported as the top 3 most concerning organisms. *C. difficile* and ESBL were reported as common or very common by most respondents, but CRE was considered rare by most respondents. Therefore, prevalence was not the only driver of concern. Generally, CRE and ESBL are not cohorted in shared rooms owing to the variety of pathogens and resistance mechanisms encompassed, and as survey responses showed, once CP are implemented for CRE, they are rarely cleared. Therefore, there may be operational pressures to modify the use of CP, AS, or both. Interpretation is conjecture, however, since the reasons for organisms selected as most concerning were not further detailed.

This survey represents a small sample size limiting generalizability. Respondents were disproportionately from large facilities located in cities, despite attempts to engage more rural participants. Academic hospitals were also over-represented as evidenced by the large majority of respondents who identified as non-government, not-for-profit, and teaching institutions. The inclusion of CHES in this survey, however, bolstered representation of community hospitals. These demographics, in general, reflect the composition of the SRN and are similar to the demographics reported in other SRN surveys. Furthermore, representation from 25 states, encompassing a wide range of practices, significantly enhances the understanding of infection prevention and control strategies being employed across the United States.

A limitation of the branching logic used in the survey is the conditional exposure to follow-up questions. Facilities who reported not using a particular practice were not asked additional questions about that practice. Nonresponses or skipped questions could have contributed to under-representation of some practices and limited robustness of conclusions drawn from those data. This limitation was counterbalanced by the detailed information that was derived regarding endorsed practices.

In summary, this survey found that infection control practices for MDRO continue to evolve. There continues to be significant institutional variability in AS practices and the application and discontinuation of CP. Approaches vary by infection status and MDRO. Compared to prior surveys, there is a trend toward time-based clearance of CP and less microbiologic screening-based clearance. CP clearance protocols appear to be more common and are expanding to include some Gram-negative organisms, although most facilities do not employ CP clearance protocols for emerging MDRO. Endorsed practices not only differ by protocols across sites, but apparent contradictions exist within described practices. Variation may indicate the need for more data on transmission risk by disease state, organism, and time. Evidence-based strategies that allow for facility-specific choice are needed to guide MDRO prioritization, CP use and duration, and CP clearance policies, including time- or test-based protocols. Guidance should acknowledge limitations in adherence, number of private rooms and persistence of environmental reservoirs as major barriers to MDRO infection prevention and control identified in this survey.

## Supporting information

Coffey et al. supplementary material Coffey et al. supplementary material

## Supplementary material

The supplementary material for this article can be found at https://doi.org/10.1017/ice.2025.10394.

## Manuscript preparation

CARB-X provided assistance with study design and editing the manuscript.
