# Supplementary material for: Current infection control practices for multidrug-resistant organisms (MDRO): a survey of the Society for Healthcare Epidemiology of America (SHEA) research network and affiliated US-based hospitals
Source: Infect Control Hosp Epidemiol. 2026 Feb 18;47(4):386–93. doi: 10.1017/ice.2025.10394 (PMC13216800; doi:10.1017/ice.2025.10394)
Supplement: Coffey et al. supplementary material [file S0899823X25103942sup001.pdf]

USE OF DECOLONIZATION STRATEGIES, AND ISOLATION/DE-ISOLATION POLICIES IN UNITED STATES HEALTHCARE FACILITIES

InstructionsThis survey should take 30 minutes to complete. Please answer the questions in sequential order. The survey tool will use "skip logic" to move you to pertinent questions based on your responses. Participation is completely voluntary. By entering information into the survey responses, you are confirming your voluntary agreement to participate in this survey, understanding your responses will be kept confidential and you are free to withdraw at any time without penalty. Please carefully read the information provided before beginning. You may save your responses and return later, but once submitted, you will not be permitted to submit additional responses.Instructions for Respondents from Healthcare SystemsIf you are answering on behalf of a healthcare system that has multiple locations and/or levels of care, please answer facility-specific questions based on one facility that best represents the overall practices of the system as a whole.   If you ever encounter an acronym that you would like more information about, please use the helper button in the upper right hand corner to review their meanings.
If you have any questions or need assistance completing this survey, please contact:KC Coffey, MD, MPHKaren.coffey@som.umaryland.eduYour participation will help guide future activities to analyze the impact of MDRO screening, decolonization practices, isolation/de-isolation strategies and is essential to the improved understanding of how these activities impact nosocomial infection rates. Thank you for your input

Demographics

If your site is part of a larger healthcare system that has multiple locations and/or levels of care, please answer subsequent facility-specific questions based on one facility that best represents the practices of the system as a whole.

____

SRN ID

- If you are a member of Community Healthcare Epidemiologists and Stewards, enter CHES
- If you do not know your SRN ID or are not an SRN member enter UNK

____

In which state or territory is your facility located?

◯ Alabama ◯ Alaska ◯ Arizona ◯ Arkansas ◯ California ◯ Colorado ◯ Connecticut
◯ Delaware ◯ District of Columbia(DC) ◯ Florida ◯ Georgia ◯ Hawaii ◯ Idaho
◯ Illinois ◯ Indiana ◯ Iowa ◯ Kansas ◯ Kentucky ◯ Louisiana ◯ Maine ◯ Maryland
◯ Massachusetts ◯ Michigan ◯ Minnesota ◯ Mississippi ◯ Missouri ◯ Montana
◯ Nebraska ◯ Nevada ◯ New Hampshire ◯ New Jersey ◯ New Mexico ◯ New York
◯ North Carolina ◯ North Dakota ◯ Ohio ◯ Oklahoma ◯ Oregon ◯ Pennsylvania
◯ Rhode Island ◯ South Carolina ◯ South Dakota ◯ Tennessee ◯ Texas ◯ Utah
◯ Vermont ◯ Virginia ◯ Washington ◯ West Virginia ◯ Wisconsin ◯ Wyoming
◯ American Somoa ◯ GUAM ◯ Northern Mariana Islands ◯ Puerto Rico ◯ US Virgin Islands

---

How would you describe the area in which your facility is located?

◯ Rural
◯ Small town, population < 20,000
◯ Town, population 20,000-49,999
◯ Small city, population 50,000-250,000
◯ Large city, population >250,000

Which of the following best describes your facility? (Choose only one)

◯ Government, nonfederal (i.e., state, county, city, etc.)
◯ Investor-owned, for-profit
◯ Nongovernment, not-for-profit
◯ Government, federal (i.e., Veteran's affairs)

Would your facility be considered a teaching institution (e.g., does your facility have medical residents, interns, or other healthcare trainees)?

◯ Yes {teaching_detail}
◯ No

How many licensed beds does your facility have?

◯ Less than 50
◯ 50-99
◯ 100-199
◯ 200-299
◯ 300-399
◯ 400-499
◯ 500-599
◯ 600-699
◯ 700-799
◯ 800 or more

Is your facility considered a Medicare Critical Access Hospital?

◯ Yes ◯ No

Does your facility have an Intensive Care Unit?

◯ Yes ◯ No

Does your facility serve a pediatric only or predominantly pediatric population?

◯ Yes ◯ No

How many admissions does your facility have each year, on average?

◯ Less than 1000
◯ 1000-4999
◯ 5000-9,999
◯ 10,000-49,999
◯ 50,000 or more
◯ I don't know the number of admissions per year

What kinds of rooms are available in your facility?

◯ Predominantly single occupancy
◯ Predominantly double occupancy
◯ Mix of multiple shared room types, i.e., single, double, quad, and open bay

MDRO Practices

Multi-Drug Resistant Organism Infection Prevention and Control Practices

4%

Please answer the questions below in the setting of routine Infection Prevention and Control practices NOT additional steps taken in the setting of an outbreak response.

Does your facility place ANY patients infected or colonized with any of the following MDRO on contact precautions as defined by the Centers for Disease Control and Prevention (CDC)?

Organism
MRSA Infection ______
Colonization ______
VRE Infection ______
Colonization ______
ESBL Infection ______
Colonization ______
CRE Infection ______
Colonization ______
MDR-PsA Infection ______
Colonization ______
CR-PsA Infection ______
Colonization ______
MDR-AB Infection ______
Colonization ______
CRAB Infection ______
Colonization ______
C. difficile Infection ______
Colonization ______
______ Infection ______
Colonization ______

Which patient populations with those MDRO are placed on contact precautions?

	All	ICU	Immune Compromised(Transplant, Oncology)	Surgical	Other
MRSA	☐	☐	☐	☐	☐
VRE	☐	☐	☐	☐	☐
ESBL	☐	☐	☐	☐	☐
CRE	☐	☐	☐	☐	☐
MDR-PsA	☐	☐	☐	☐	☐
CR-PsA	☐	☐	☐	☐	☐
MDR-AB	☐	☐	☐	☐	☐

CRAB	☐	☐	☐	☐	☐
C. difficile	☐	☐	☐	☐	☐
[place_organism_other]	☐	☐	☐	☐	☐

What other patient populations with MRSA are placed on contact precautions?

What other patient populations with VRE are placed on contact precautions?

What other patient populations with ESBL are placed on contact precautions?

What other patient populations with CRE are placed on contact precautions?

What other patient populations with MDR-PsA are placed on contact precautions?

What other patient populations with CR-PsA are placed on contact precautions?

What other patient populations withMDR-AB are placed on contact precautions?

What other patient populations with CRAB are placed on contact precautions?

What other patient populations with C. difficile are placed on contact precautions?

What other patient populations with [place_organism_other] are placed on contact precautions?

If a patient with one of the following MDRO is discharged from your facility and then readmitted, are contact precautions reinstituted based on the information from the prior admission?

	Yes, for all admissions	Yes, until criteria for de-isolation are met	No
MRSA	○	○	○
VRE	○	○	○
ESBL	○	○	○
CRE	○	○	○
MDR-PsA	○	○	○
CR-PsA	○	○	○
MDR-AB	○	○	○
CRAB	○	○	○
C. difficile	○	○	○
[place_organism_other]	○	○	○

Are any additional measures utilized for patients with the above MDROs to prevent transmission?

	Signage	Enhanced room cleaning during stay	Enhanced room cleaning after discharge	Additional cleaning of reusable medical equipment	Cohorting patients	Cohorting healthcare staff	Other
MRSA	☐	☐	☐	☐	☐	☐	☐
VRE	☐	☐	☐	☐	☐	☐	☐
ESBL	☐	☐	☐	☐	☐	☐	☐
CRE	☐	☐	☐	☐	☐	☐	☐
MDR-PsA	☐	☐	☐	☐	☐	☐	☐
CR-PsA	☐	☐	☐	☐	☐	☐	☐
MDR-AB	☐	☐	☐	☐	☐	☐	☐
CRAB	☐	☐	☐	☐	☐	☐	☐
C. difficile	☐	☐	☐	☐	☐	☐	☐
[place_organism_other]	☐	☐	☐	☐	☐	☐	☐

Barriers to MDRO infection Prevention

What are the top 3 barriers to MDRO Infection Prevention in your facility?
(Choose 3)

- ☐ Training/adherence frontline staff
- ☐ Availability micro/diagnostic services
- ☐ Access to HAI data
- ☐ Communication between healthcare systems
- ☐ Insufficient support from unit management
- ☐ Insufficient administrative support
- ☐ Insufficient supplies (personal protective equipment, alcohol-based hand rub)
- ☐ Insufficient staffing ______
- ☐ Access to decolonization/deisolation products
- ☐ Availability of private rooms
- ☐ Access to real-time data, informatics, or other technology
- ☐ Lack of standardized environmental testing
- ☐ Lack of evidence-based strategies to eradicate environmental reservoirs (example, sinks)
- ☐ Other ______

MDRO Surveillance

Surveillance Practices

20%

Please answer the questions below in the setting of routine Surveillance practices NOT additional steps taken in the setting of an outbreak response.
Does your facility perform active surveillance of any patient population for any of the following MDRO?

	Yes	No
MRSA	◯	◯
VRE	◯	◯
ESBL	◯	◯
CRE	◯	◯
MDR-PsA	◯	◯
CR-PsA	◯	◯
MDR-AB	◯	◯
CRAB	◯	◯
C. difficile	◯	◯
[place_organism_other]	◯	◯

For the organisms identified as undergoing active surveillance (AS), is AS mandated by legislation?

	Yes	No
MRSA	◯	◯
VRE	◯	◯
ESBL	◯	◯
CRE	◯	◯
MDR-PsA	◯	◯
CR-PsA	◯	◯
MDR-AB	◯	◯
CRAB	◯	◯
C. difficile	◯	◯
[place_organism_other]	◯	◯

When do you perform active surveillance to identify asymptomatic colonization? Check all that apply

	On all admissions	On admissions to specific units	At intervals throughout admission	On discharge from a specific unit	On discharge from the facility
MRSA	☐	☐	☐	☐	☐
VRE	☐	☐	☐	☐	☐
ESBL	☐	☐	☐	☐	☐
CRE	☐	☐	☐	☐	☐
MDR-PsA	☐	☐	☐	☐	☐
CR-PsA	☐	☐	☐	☐	☐
MDR-AB	☐	☐	☐	☐	☐
CRAB	☐	☐	☐	☐	☐
C. difficile	☐	☐	☐	☐	☐
[place_organism_other]	☐	☐	☐	☐	☐

For which patients do you perform active surveillance to identify asymptomatic colonization?

	All	ICU	Immune compromised (Transplant,	Surgical	Point Prevalence Survey(s)	Other
MRSA	☐	☐	☐	☐	☐	☐
VRE	☐	☐	☐	☐	☐	☐
ESBL	☐	☐	☐	☐	☐	☐
CRE	☐	☐	☐	☐	☐	☐
MDR-PsA	☐	☐	☐	☐	☐	☐
CR-PsA	☐	☐	☐	☐	☐	☐
MDR-AB	☐	☐	☐	☐	☐	☐
CRAB	☐	☐	☐	☐	☐	☐
C. difficile	☐	☐	☐	☐	☐	☐
[place_organism_other]	☐	☐	☐	☐	☐	☐

Microbiology Laboratory Capacity
For each of the MDROs for which active surveillance is performed, where is this laboratory testing performed

	On-Site	Off-site within the system	Send out-commerci al lab	Send out-state lab	Send out-reference lab	Other
MRSA	☐	☐	☐	☐	☐	☐
VRE	☐	☐	☐	☐	☐	☐
ESBL	☐	☐	☐	☐	☐	☐
CRE	☐	☐	☐	☐	☐	☐
MDR-PsA	☐	☐	☐	☐	☐	☐

CR-PsA	☐	☐	☐	☐	☐	☐
MDR-AB	☐	☐	☐	☐	☐	☐
CRAB	☐	☐	☐	☐	☐	☐
C. difficile	☐	☐	☐	☐	☐	☐
[place_organism_other]	☐	☐	☐	☐	☐	☐

For each of the targeted MDROs for which active surveillance is performed, what is the usual time from collection to result?

	< 24 Hours	24 Hours to < 48 Hours	48 Hours to < 1 Week	> 1 Week	Do not know
MRSA	○	○	○	○	○
VRE	○	○	○	○	○
ESBL	○	○	○	○	○
CRE	○	○	○	○	○
MDR-PsA	○	○	○	○	○
CR-PsA	○	○	○	○	○
MDR-AB	○	○	○	○	○
CRAB	○	○	○	○	○
C. difficile	○	○	○	○	○
[place_organism_other]	○	○	○	○	○

For each of the targeted MDROs for which active surveillance is performed, what type of assay is performed?

	Culture	PCR-single pathogen	PCR-multiplex assay	Other	Don't Know
MRSA	☐	☐	☐	☐	☐
VRE	☐	☐	☐	☐	☐
ESBL	☐	☐	☐	☐	☐
CRE	☐	☐	☐	☐	☐
MDR-PsA	☐	☐	☐	☐	☐
CR-PsA	☐	☐	☐	☐	☐
MDR-AB	☐	☐	☐	☐	☐
CRAB	☐	☐	☐	☐	☐
C. difficile	☐	☐	☐	☐	☐
[place_organism_other]	☐	☐	☐	☐	☐

What other type of assay is performed?

MDRO Isolation Practices

Isolation Practices

40%

Do you implement pre-emptive or empiric contact precautions for certain patient populations WITHOUT known MDRO who are admitted or transferred to your facility?

MRSA	◯ No	◯ Yes
VRE	◯ No	◯ Yes
ESBL	◯ No	◯ Yes
CRE	◯ No	◯ Yes
MDR-PsA	◯ No	◯ Yes
CR-PsA	◯ No	◯ Yes
MDR-AB	◯ No	◯ Yes
CRAB	◯ No	◯ Yes
C. difficile	◯ No	◯ Yes
[place_organism_other]	◯ No	◯ Yes

If employing pre-emptive or empiric contact precautions, what factors determine whether isolation is implemented? (Check all that apply)

- ☐ Direct transfer from a facility with known outbreak of a targeted MDRO
- ☐ Transfer from or recent hospitalization in a facility in a specified U.S. region
- ☐ Transfer from or recent hospitalization in a facility in a specified non-U.S. region
- ☐ Transfer from any long-term care facility
- ☐ Other risk factors ______
- ☐ Don't know

What is the relative frequency of the following MDRO in your facility?

	Rare	Common	Very Common	Don't Know
MRSA	◯	◯	◯	◯
VRE	◯	◯	◯	◯
ESBL	◯	◯	◯	◯

CRE	○	○	○	○
MDR-PsA	○	○	○	○
CR-PsA	○	○	○	○
MDR-AB	○	○	○	○
CRAB	○	○	○	○
C. difficile	○	○	○	○
[place_organism_other]	○	○	○	○

Which 3 MDRO are the most troubling in your facility?
(Choose 3)

- ☐ MRSA
- ☐ VRE
- ☐ ESBL
- ☐ CRE
- ☐ MDR-PsA
- ☐ CR-PsA
- ☐ MDR-AB
- ☐ CRAB
- ☐ C. difficile
- ☐ [place_organism_other]

MDRO Deisolation Practices

De-Isolation Practices

47%

Does your facility have a protocol for discontinuing contact precautions for patients with a history of any of the following MDRO?

	No (once identified as having this MDRO, they remain on precautions in my facility for life)	Yes	I don't know
MRSA	◯	◯	◯
VRE	◯	◯	◯
ESBL	◯	◯	◯
CRE	◯	◯	◯
MDR-PsA	◯	◯	◯
CR-PsA	◯	◯	◯
MDR-AB	◯	◯	◯
CRAB	◯	◯	◯
C. difficile	◯	◯	◯
[place_organism_other]	◯	◯	◯

If your facility has a protocol for discontinuation of contact precautions for patients with a history of these MDRO, please indicate which of the following are components of the protocol.

	Time since last positive test	Microbiological confirmation of clearance	Other
MRSA	☐	☐	☐
VRE	☐	☐	☐
ESBL	☐	☐	☐
CRE	☐	☐	☐
MDR-PsA	☐	☐	☐
CR-PsA	☐	☐	☐
MDR-AB	☐	☐	☐
CRAB	☐	☐	☐
C. difficile	☐	☐	☐
[place_organism_other]	☐	☐	☐

What other components are part of the protocol?

Please tell us how much time must elapse for a patient to be considered for discontinuation of contact precautions.

	End on Discharge	Less than one month	1-3 Months	>3-6 Months	>6-12 Months	More than one year
MRSA criteria	○	○	○	○	○	○
VRE criteria	○	○	○	○	○	○
ESBL criteria	○	○	○	○	○	○
CRE criteria	○	○	○	○	○	○
MDR-PsA criteria	○	○	○	○	○	○
CR-PsA criteria	○	○	○	○	○	○
MDR-AB criteria	○	○	○	○	○	○
CRAB criteria	○	○	○	○	○	○
C. difficile criteria	○	○	○	○	○	○
other	○	○	○	○	○	○

If your facility's MDRO protocol includes microbiological testing to document clearance, which sites are sampled and how many specimens must be obtained? Please check all that apply.

Organism Site(s) Total Number of Specimens per Patient

MRSA ______ ______

VRE ______ ______

VRE Clearance Testing

____

ESBL ______ ______

ESBL Clearance Testing Other

____

CRE ______ ______

CRE Clearance Testing Other

____

MDR-PsA ______ ______

MDR-PsA Clearance Testing Other

____

CR-PsA ______ ______

CR-PsA Clearance Testing Other

____

MDR-AB ______ ______

MDR-AB Clearance Testing Other __________________________________

CRAB ______ ______

CRAB Clearance Testing Other __________________________________

C. difficile ______ ______

CDIFF Clearance Testing Other __________________________________

other ______ ______

Other Clearance Testing Other __________________________________

If your facility's MDRO protocol includes more than one microbiological test from a body site to document clearance, how much time must elapse between sampling?

	< 24 hours	24-48 hours	>48 hours - 1 week	>1 week	Other
MRSA	○	○	○	○	○
VRE	○	○	○	○	○
ESBL	○	○	○	○	○
CRE	○	○	○	○	○
MDR-PsA	○	○	○	○	○
CR-PsA	○	○	○	○	○
MDR-AB	○	○	○	○	○
CRAB	○	○	○	○	○
C. difficile	○	○	○	○	○
other	○	○	○	○	○

Please detail what other time elapsed your facility uses

Concurrent Antibiotics

If your facility's MDRO protocol includes microbiological testing to document clearance, does concurrent use of antibiotics or decolonization agents affect timing of microbiologic testing?

○ Yes ______
○ No

MDRO Decolonization Practices

Decolonization Practices

68%

Does your facility currently employ any form of decolonization? ◯ Yes ◯ No

Which outcome is most important when considering whether or not to implement a targeted decolonization policy?

◯ Lab-based clearance documentation
◯ Significant reduction in risk of transmission to other patients
◯ Significant risk reduction for the colonized patient progression to infection

What decolonization practices does your facility employ? (Check all that apply)

☐ Topical antiseptic (e.g., chlorhexidine, iodophor)
☐ Topical antibiotic (e.g., mupirocin)
☐ Oral non-absorbed antibiotic (e.g., oral vancomycin, rifaximin)
☐ Oral absorbed antibiotic (e.g., , doxycycline, rifampin)
☐ Enteral non-antibiotic biologic (e.g., fecal microbiota or probiotics)
☐ Parenteral (IV) antibiotic
☐ None of these

Are certain patient populations targeted for decolonization? ◯ Yes ◯ No

If certain patient populations are targeted for decolonization, which patient populations?

☐ All______
☐ ICU______
☐ Transplant/oncology______
☐ Transfers______
☐ Contacts during outbreak investigation______
☐ Surgical______
☐ Other ____________

Does your facility target specific MDROs for decolonization? ◯ Yes ◯ No

[place_organism_other_helper] ______________________________

MRSA decolonization ◯ Yes ◯ No

MRSA Decolonization Target Detail

☐ Topical antiseptic
☐ Topical antibiotic
☐ Oral (non-absorbed) antibiotic
☐ Oral absorbed antibiotic
☐ Enteral biologic (e.g., fecal microbiota or probiotics)
☐ Parenteral (IV) antibiotic

VRE decolonization ◯ Yes ◯ No

VRE Decolonization Target Detail

☐ Topical antiseptic
☐ Topical antibiotic
☐ Oral (non-absorbed) antibiotic
☐ Oral absorbed antibiotic
☐ Enteral biologic (e.g., fecal microbiota or probiotics)
☐ Parenteral (IV) antibiotic

ESBL decolonization ◯ Yes ◯ No

ESBL Decolonization Target Detail

☐ Topical antiseptic
☐ Topical antibiotic
☐ Oral (non-absorbed) antibiotic
☐ Oral absorbed antibiotic
☐ Enteral biologic (e.g., fecal microbiota or probiotics)
☐ Parenteral (IV) antibiotic

CRE decolonization ◯ Yes ◯ No

CRE Decolonization Target Detail

☐ Topical antiseptic
☐ Topical antibiotic
☐ Oral (non-absorbed) antibiotic
☐ Oral absorbed antibiotic
☐ Enteral biologic (e.g., fecal microbiota or probiotics)
☐ Parenteral (IV) antibiotic

MDR-PsA decolonization ◯ Yes ◯ No

MDR-PsA Decolonization Target Detail

☐ Topical antiseptic
☐ Topical antibiotic
☐ Oral (non-absorbed) antibiotic
☐ Oral absorbed antibiotic
☐ Enteral biologic (e.g., fecal microbiota or probiotics)
☐ Parenteral (IV) antibiotic

CR-PsA decolonization ◯ Yes ◯ No

CR-PsA Decolonization Target Detail

☐ Topical antiseptic
☐ Topical antibiotic
☐ Oral (non-absorbed) antibiotic
☐ Oral absorbed antibiotic
☐ Enteral biologic (e.g., fecal microbiota or probiotics)
☐ Parenteral (IV) antibiotic

MDR-AB decolonization ◯ Yes ◯ No

MDR-AB Decolonization Target Detail

☐ Topical antiseptic
☐ Topical antibiotic
☐ Oral (non-absorbed) antibiotic
☐ Oral absorbed antibiotic
☐ Enteral biologic (e.g., fecal microbiota or probiotics)
☐ Parenteral (IV) antibiotic

CRAB decolonization ◯ Yes ◯ No

CRAB Decolonization Target Detail

☐ Topical antiseptic
☐ Topical antibiotic
☐ Oral (non-absorbed) antibiotic
☐ Oral absorbed antibiotic
☐ Enteral biologic (e.g., fecal microbiota or probiotics)
☐ Parenteral (IV) antibiotic

C. difficile decolonization ◯ Yes ◯ No

C. difficile Decolonization Target Detail

☐ Topical antiseptic
☐ Topical antibiotic
☐ Oral (non-absorbed) antibiotic
☐ Oral absorbed antibiotic
☐ Enteral biologic (e.g., fecal microbiota or probiotics)
☐ Parenteral (IV) antibiotic

other decolonization ◯ Yes ◯ No

other Decolonization Target Detail

☐ Topical antiseptic
☐ Topical antibiotic
☐ Oral (non-absorbed) antibiotic
☐ Oral absorbed antibiotic
☐ Enteral biologic (e.g., fecal microbiota or probiotics)
☐ Parenteral (IV) antibiotic

For each MDRO undergoing decolonization, what is/are the anatomical sites for decolonization?

	Decolonization bathing	Nares	Oropharynx	Groin	Axilla	Site of original clinical infection (e.g., wound)	Enteral	Other
MRSA	☐	☐	☐	☐	☐	☐	☐	☐
VRE	☐	☐	☐	☐	☐	☐	☐	☐
ESBL	☐	☐	☐	☐	☐	☐	☐	☐
CRE	☐	☐	☐	☐	☐	☐	☐	☐
MDR-PsA	☐	☐	☐	☐	☐	☐	☐	☐
CR-PsA	☐	☐	☐	☐	☐	☐	☐	☐
MDR-AB	☐	☐	☐	☐	☐	☐	☐	☐
CRAB	☐	☐	☐	☐	☐	☐	☐	☐
C. difficile	☐	☐	☐	☐	☐	☐	☐	☐
[place_organism_other]	☐	☐	☐	☐	☐	☐	☐	☐

For those MDRO for which decolonization is performed, if patients were on contact precautions are they de-isolated (contact precautions removed) following decolonization?

	Yes	No	N/A
MRSA	○	○	○
VRE	○	○	○
ESBL	○	○	○
CRE	○	○	○
MDR-PsA	○	○	○
CR-PsA	○	○	○
MDR-AB	○	○	○
CRAB	○	○	○
C. difficile	○	○	○
[place_organism_other]	○	○	○

MDRO Needs Or Gaps

Needs or Gaps

81%

In this section, you will be presented with a series of short scenarios aimed at assessing how your decolonization product preferences vary by clinical indicators. (Hover over answer choices for additional information.)

A 65-year-old man presents to your facility with chest pain and will be admitted. Prior to admission, he grew CRE in a urine culture. He has no urinary symptoms now.

If this patient were being admitted to a non-ICU unit, would you want to decolonize this patient for infection control purposes?

◯ Yes ◯ No

Why wouldn't you want to decolonize this patient?

◯ Wouldn't protect the patient
◯ Wouldn't change transmission
◯ Wouldn't work in time
◯ Effect won't last
◯ Efficacy not worth the effort
◯ Patients don't like decolonization
◯ Likely too broad or too narrow
◯ Product will alter normal flora
◯ Too costly
◯ Other ______

If you were to decolonize this patient, choose the top three decolonization agent characteristics that would meet your patient's needs.
(Please select only 3)

☐ Patient-specific efficacy
☐ Transmission interruption
☐ Time to onset
☐ Durability
☐ Ease
☐ Tolerability
☐ Breadth
☐ Impact on the microbiome
☐ Cost
☐ Other ______

If the same patient were being admitted to an ICU, would you want to be able to decolonize this patient?

◯ Yes ◯ No

Why wouldn't you want to decolonize this patient?

- ◯ Wouldn't protect the patient
- ◯ Wouldn't change transmission
- ◯ Wouldn't work in time
- ◯ Effect won't last
- ◯ Efficacy not worth the effort
- ◯ Patients don't like decolonization
- ◯ Likely too broad or too narrow
- ◯ Product will alter normal flora
- ◯ Too costly
- ◯ Other ______

If you were to decolonize this patient, choose the top three decolonization agent characteristics that would meet your patient's needs.
(Please select only 3)

- ☐ Patient-specific efficacy
- ☐ Transmission interruption
- ☐ Time to onset
- ☐ Durability
- ☐ Ease
- ☐ Tolerability
- ☐ Breadth
- ☐ Impact on the microbiome
- ☐ Cost
- ☐ Other ______

If the same patient were scheduled for coronary artery bypass graft (CABG) surgery, would you want to be able to decolonize this patient? ◯ Yes ◯ No

Why wouldn't you want to decolonize this patient?

- ◯ Wouldn't protect the patient
- ◯ Wouldn't change transmission
- ◯ Wouldn't work in time
- ◯ Effect won't last
- ◯ Efficacy not worth the effort
- ◯ Patients don't like decolonization
- ◯ Likely too broad or too narrow
- ◯ Product will alter normal flora
- ◯ Too costly
- ◯ Other ______

If you were to decolonize this patient, choose the top three decolonization agent characteristics that would meet your patient's needs.
(Please select only 3)

- ☐ Patient-specific efficacy
- ☐ Transmission interruption
- ☐ Time to onset
- ☐ Durability
- ☐ Ease
- ☐ Tolerability
- ☐ Breadth
- ☐ Impact on the microbiome
- ☐ Cost
- ☐ Other ______

If this same patient were a recent heart transplant recipient, would you want to be able to decolonize this patient?

◯ Yes ◯ No

Why wouldn't you want to decolonize this patient?

◯ Wouldn't protect the patient
◯ Wouldn't change transmission
◯ Wouldn't work in time
◯ Effect won't last
◯ Efficacy not worth the effort
◯ Patients don't like decolonization
◯ Likely too broad or too narrow
◯ Product will alter normal flora
◯ Too costly
◯ Other ______

If you were to decolonize this patient, choose the top three decolonization agent characteristics that would meet your patient's needs.
(Please select only 3)

☐ Patient-specific efficacy
☐ Transmission interruption
☐ Time to onset
☐ Durability
☐ Ease
☐ Tolerability
☐ Breadth
☐ Impact on the microbiome
☐ Cost
☐ Other ______

Emerging Infection

The Centers for Disease Control and Prevention (CDC) has identified a new, emerging pathogen found to cause healthcare-associated infections, but no existing antiseptics or antibiotics have any proven efficacy in preventing transmission or progression from colonization to infection.

If the following novel agents became available (FDA approved) and were effective for decolonization of the skin and/or gastrointestinal tract, which mode would you be most interested in trying? Score as low (not interested), medium (no preference) or high (very interested).

Agent Rating Why?
New topical antiseptic

______ ______
New topical antimicrobial

______ ______
New oral (non-absorbed) antibiotic

______ ______
New oral (absorbed) antibiotic

______ ______
New parenteral (IV) antibiotic

______ ______
New enteral biologic (e.g., fecal microbiota, probiotic, or live biotherapeutic product)

______ ______
Phage therapy

(a virus that targets and kills the new pathogen)

______ ______ ______

How would your interest change once the emerging pathogen became endemic in the community, e.g. >5% of new admissions are colonized with the new pathogen? Agent Rating Why?

New topical antiseptic

______ ______

New topical antimicrobial

______ ______

New oral (non-absorbed) antibiotic

______ ______

New oral (absorbed) antibiotic

______ ______

New parenteral (IV) antibiotic

______ ______

New enteral biologic (e.g., fecal microbiota, probiotic, or live biotherapeutic product)

______ ______

Phage therapy

(a virus that targets and kills the new pathogen)

______ ______

______ ______ ______

If this new pathogen becomes endemic, what are the top three considerations in implementing a targeted decolonization campaign?
(Select only 3)

- ☐ Patient-specific efficacy
- ☐ Transmission interruption
- ☐ Time to onset
- ☐ Durability
- ☐ Ease
- ☐ Tolerability
- ☐ Breadth
- ☐ Impact on the microbiome
- ☐ Cost
- ☐ Other ______

If this new pathogen was rare, but led to severe disease, what would be the top three considerations in implementing a targeted decolonization campaign?
(Select only 3)

- ☐ Patient-specific efficacy
- ☐ Transmission interruption
- ☐ Time to onset
- ☐ Durability
- ☐ Ease
- ☐ Tolerability
- ☐ Breadth
- ☐ Impact on the microbiome
- ☐ Cost
- ☐ Other ______

Does your facility have any unmet decolonization needs? ◯ Yes ◯ No

---

Gram positive MDRO Needs

Gram negative MDRO Needs
